# Construction of a three-level enteral nutrition nursing system under the “Internet + medical” mode and an evaluation of its effect in clinical application

**DOI:** 10.3389/fpubh.2022.976276

**Published:** 2022-09-27

**Authors:** Min Hu, Yan Ling, Fang-Ting Xiong, Jian-Mei Xu

**Affiliations:** ^1^Department of Neurology, The Second Affiliated Hospital of Nanchang University, Nanchang, China; ^2^Department of Neurosurgery, The Second Affiliated Hospital of Nanchang University, Nanchang, China

**Keywords:** Internet, medical care, enteral nutrition, management platform, nursing

## Abstract

**Objective:**

This study aimed to explore the construction of a three-level enteral nutrition nursing system under the “Internet + medical” mode and the clinical application effect.

**Methods:**

A total of 40 nurses from four primary and secondary hospitals in Jiangxi Province and 80 patients treated with enteral nutrition between January 2020 and December 2021 were enrolled in this study. Patients in the control group received routine enteral nutrition nursing. In the study group, a three-level enteral nutrition nursing system was applied under the “Internet + medical” mode to train and guide the implementation of clinical enteral nutrition. The changes in nurses' cognition and behavior in enteral nutrition safety nursing, comprehensive core competence before and after training, and the effect of enteral nutrition nursing were compared between the two groups.

**Results:**

After 3 months of training, nurses' cognition and behavior scores in enteral nutrition safety nursing were higher than those before training (*t* = 11.780, *P* < 0.05), and nurses' core competence scores were higher than before training (*P* < 0.05). After 1 week of nursing, the nutritional risk screening 2002 (NRS2002) score decreased, and the levels of albumin and hemoglobin increased in both groups (*P* < 0.05). However, after 1 week of nursing, the NRS2002 score of the study group (2.89 ± 0.75) was lower than that of the control group (3.25 ± 0.82), and the levels of albumin (39.89 ± 3.21) and hemoglobin (119.57 ± 8.78) were higher in the study group than in the control group (albumin 36.25 ± 3.45, hemoglobin 113.66 ± 9.55) (*P* < 0.05).

**Conclusion:**

Three-level enteral nutrition nursing linkage assisted by the “Internet + medical” mode can improve the cognition and behavior of medical staff in enteral nutrition safety nursing, as well as the comprehensive core competence of nurses, achieving good clinical effects.

## Introduction

Enteral nutrition (oral nutrition + tube feeding nutrition), as the first choice in clinical nutritional treatment, can effectively improve the nutritional status of patients, reduce the occurrence of complications, and decrease the length of hospital stays (1, 2). Clinical reports show that, due to the depletion of diseases and patients' insufficient nutritional intake, the number of patients with malnutrition is increasing (3). However, through the construction of a normalized and standardized enteral nutrition nursing system, professional services can be provided for clinical nutrition evaluation, implementation, observation, nursing, and follow-up family nutrition management, which can greatly improve the effect of nutritional treatment (4, 5).

Primary and secondary hospitals at the grass-roots level are important hubs of China's health service network and play an irreplaceable role in local health work; however, due to the limitation of medical resources, the nursing level of enteral nutrition in such hospitals has fallen behind that of other centers. To address this, the present study used the “Internet + medical” mode and the practical experience of the multidisciplinary enteral nutrition nursing team of our hospital to develop a three-level enteral nutrition management system assisted by the “Internet + medical” mode. It is hoped that this will provide a reference for the training of grass-roots nursing staff and the construction of medical union.

## Subjects and methods

### Subjects

#### Patients

A total of 80 patients in need of enteral nutrition treatment admitted from January 2020 to December 2021 were selected as the research objects. These data were obtained from four primary and secondary hospitals.

Inclusion criteria: (1) Patient age >18 years; (2) indication of enteral nutrition; (3) nutritional risk screening 2002 (NRS2002) score >3; (4) normal cognitive ability; (5) hospital stay >1 week.

Exclusion criteria: (1) The presence of intestinal diseases, such as intestinal ischemia, intestinal obstruction, and intestinal perforation; (2) patients with very severe craniocerebral injury or patients at a terminal stage of disease with a short life expectancy.

A total of 40 patients from January to December 2020 were selected as the control group, and 40 patients from January to December 2021 were selected as the study group. There were 21 male patients and 19 female patients in the control group, with an age range of 18–75 years (average age 43.58 ± 10.53 years). The cases in the control group included 15 cases of cerebral infarction, 12 cases of acute pancreatitis, 6 cases of tumor, and seven cases of craniocerebral trauma. There were 23 female patients and 17 male patients in the study group, with an age range of 18–75 years (average age 44.25 ± 9.86 years). The cases in the study group included 13 cases of cerebral infarction, 3 cases of tumor, 12 cases of craniocerebral trauma, and 12 cases of acute pancreatitis. The general data of the two groups were comparable (*P* > 0.05).

#### Medical staff

A total of 40 clinical nutrition nurses (37 females and three males) from primary and secondary grass-roots hospitals were enrolled as training subjects. The age range of the nurses was 25–48 years, with an average age of 33.68 ± 5.74 years, and their working life was 6–15 years, with an average of 7.82 ± 2.11 years. A total of 32 nurses were educated to junior college level or below, while eight of the nurses were educated to undergraduate level or above. Two of the nurses were chief nurses, eight were deputy chief nurses, 11 were supervisory nurses, and 19 were nurses.

### Research methods

The patients in the study group received nursing using a three-level hospital enteral nutrition nursing system assisted by the “Internet + medical” mode.

Patients in the control group who received enteral nutrition nursing from January to December 2020 were selected, and conventional enteral nutrition nursing method was adopted. According to the doctor's advice, input nutritional preparations or push homemade nutrient solution, infusion at the conventional speed, when the patient has adverse reactions, slow down the speed or stop feeding according to the doctor's advice, etc.

Patients in the study group Patients who received enteral nutrition care from January to December 2021 were selected, these nurses who were trained by the enteral nutrition nursing system of the “Internet + Medical” tertiary hospital to care for patients. The details are as follows:

#### Construction of the “Internet + medical” mode three-level enteral nutrition nursing system

First, an “Internet + medical” mode three-level enteral nutrition specialist nursing technical support team was established. The team was composed of 12 members of multidisciplinary enteral nutrition nursing teams, with the head of the specialist team acting as the team leader. The team members included specialist nutrition nurses, as well as medical staff from high-risk nutrition departments, nutrition departments, pharmacy departments, and information departments. The team was mainly responsible for online training, problem consultation, platform development, and offline field guidance. All team members had worked in nursing for more than 5 years, held intermediate or higher professional titles, and had rich experience and access to excellent technology in the fields of clinical nutrition treatment, basic nursing, nursing management, nursing research and teaching, and nutrition nursing.

Second, a management training program was developed. In the early stage, the nursing team undertook a thorough evaluation of the grass-roots hospitals to investigate the enteral nutrition work being undertaken. By discussing with the nursing leaders and main members of the grass-roots hospital teams and undertaking a thorough investigation of the nutritional baseline in the hospitals, the nursing team gained an understanding of the clinical nutritional needs and integrated them with the training plan formulated by the team.

Third, an “Internet + medical” mode three-level enteral nutrition management platform was developed. The platform consisted of seven modules: a basic database, a collaborative management module, a training module, a resource database module, a remote consultation module, a case sharing and discussion module, and an effect evaluation module.

Last, offline guidance and practical training was given. Tertiary hospitals regularly dispatch staff to grass-roots hospitals to provide guidance and investigation. As such, the medical staff of primary and secondary grass-roots hospitals were accepted in batches according to the needs of each hospital and uniformly deployed to our hospital for on-the-job nutrition training, one-on-one training, teaching, and further study.

#### Implementation of the “Internet + medical” mode three-level enteral nutrition nursing system

Online training on the “Internet + medical” mode three-level enteral nutrition management platform and offline real-time guidance were provided to the 40 nurses enrolled in this study. These nurses went on to undertake clinical practice after passing the examination.

① Basic database: including medical information base and patient information base.

Medical and nursing information database: 40 medical staff who joined the enteral nutrition linkage team in primary hospitals registered and entered information through the Internet to collect personnel information, including department, name, gender, age, working years, professional title, educational background and other information.

Patient information database: Nurses in primary hospitals input patient information after training, including hospitalization number, name, gender, age, disease and other information.

② Collaborative management module: According to the status quo and needs of enteral nutrition care in primary and secondary hospitals, tertiary hospitals integrate management plans into the platform, develop personalized in-hospital enteral nutrition care work system, norms and operational process list, and organize learning. Each plan completed by primary and secondary hospitals can be uploaded through this module, and tertiary hospitals can directly supervise the completion progress of the plan online.

③ Training module: According to the formulated enteral nutrition related course arrangement, online live teaching was conducted. Theoretical lectures were conducted once a week, two classes each time and online training courses were conducted, covering nursing management, nutrition theory, nutrition norms, nutrition technology, nutrition research, etc.

④ Resource library module: The resource library includes enteral nutrition guidelines, expert consensus, relevant training videos, courseware, technical operation videos, FAQ processing flow charts and other resources, which are shared and uploaded by tertiary hospitals.

⑤ Remote consultation module: primary hospitals send some difficult clinical cases to tertiary hospitals for consultation through the remote consultation module. Tertiary hospitals, after online review, participate in online consultation to guide primary and secondary hospitals to implement enteral nutrition nursing scientifically and reasonably.

⑥ Case sharing and discussion module: select typical clinical medical records or difficult medical records, sample hospitals to participate in discussion and communication, and online Unicom video conference system at the scheduled time. Through this form, primary care staff report medical records, put forward clinical thinking, carry out diagnosis and differential diagnosis, and participate in interaction. Enteral nutrition experts in tertiary hospitals analyze and teach medical records through questions, discussions and explanations.

⑦ Effect evaluation module: nurses in primary hospitals input relevant indicators before and after the implementation of enteral nutrition in patients: name of enteral nutrition technology, number of cases, incidence of enteral nutrition-related complications, BMI index, albumin, transferrin and hemoglobin content of patients; Online assessment and questionnaire were used to evaluate and compare the cognition of enteral nutrition and nurses' self-ability evaluation before and 3 months after training.

⑧ Offline guidance and practical learning: tertiary hospital supervisors regularly go to primary hospitals for field guidance and investigation; According to the actual needs, medical staff from primary and secondary hospitals were accepted in batches to our hospital for nutrition related content rotation training, one-to-one training and teaching, and further study.

### Evaluation indexes

#### Nurses' cognition of enteral nutrition

The Cognition and Behavior Survey Scale of Enteral Nutrition Safety Nursing (6) was adopted to evaluate nurses' cognition of enteral nutrition nursing before and after 3 months of training; the total Cronbach's α coefficient for the questionnaire was 0.874. The evaluation included two parts: a cognition questionnaire and a behavior questionnaire. The cognition questionnaire of enteral nutrition safety nursing consisted of 16 questions, with a full score of 16 points; the higher the score, the better the cognition. The behavior questionnaire of enteral nutrition safety nursing consisted of 13 questions and used the Linkert four-point scoring method; the higher the score, the better the nursing behavior.

#### Comprehensive ability of nurses

The China Registered Nurse Core Competence Scale (CIRN) (7) was adopted for evaluation. The total Cronbach's α for the scale was 0.890. The scale consisted of 58 items in seven dimensions, including critical thinking/scientific research ability, clinical nursing, leadership, interpersonal relationship, ethics/legal practice, professional development, and education/consultation. The scale used Likert's five-point scoring method; the higher the total score, the better the comprehensive ability of the nurses.

#### Evaluation of nutritional nursing effect

Changes in NRS2002 (8) score, serum albumin levels, and hemoglobin levels before and after nursing were recorded.

### Statistical methods

Data were analyzed using SPSS 21.0 statistical software. Measurement data were expressed as mean ± standard deviation ($\bar{\text{x}}$ ± SD) and evaluated using a *t*-test or analysis of variance. Count data were expressed as the number of cases or a percentage (%) and evaluated using a χ^2^ test. *P* < 0.05 was considered statistically significant.

## Results

### Comparison of nurses' cognition and behavior level of enteral nutrition nursing before and after 3 months of training

After 3 months of training, the nurses' cognition and behavior scores in enteral nutrition safety nursing were higher than those before training (*P* < 0.05; see Table 1).

**Table 1:** Comparison of nurses' cognition and behavior level of enteral nutrition before and 3 months after training ($\bar{\text{x}}$ ± SD, scores).

**Index**	**Cases**	**Before training**	**After 3 months of training**	***t***	***P***
Cognition level of safety nursing	40	9.56 ± 1.89	14.78 ± 2.07	11.780	<0.001
Behavior level of safety nursing	40	5.68 ± 1.74	11.59 ± 1.98	14.180	<0.001

### Comparison of nurses' comprehensive ability scores before and after 3 months of training

After 3 months of training, the nurses' scores in critical thinking/scientific research ability, clinical nursing, leadership ability, interpersonal relationship, ethics/legal practice, professional development, and education/consultation were significantly higher than those before training (*P* < 0.05; see Table 2).

**Table 2:** Comparison of nurses' comprehensive ability scores before and 3 months after training ($\bar{x}$ ± s, scores).

**Index**	**Cases**	**Before training**	**After 3 months of training**	***t***	***P***
Critical thinking/scientific research ability	40	30.69 ± 4.15	36.53 ± 3.47	6.828	<0.001
Clinical nursing	40	28.36 ± 5.07	33.63 ± 4.32	5.004	<0.001
Leadership	40	27.93 ± 4.26	35.58 ± 4.74	7.592	<0.001
Interpersonal relationship	40	26.47 ± 3.69	29.35 ± 3.23	3.714	<0.001
Ethics/legal practice	40	20.56 ± 4.01	27.69 ± 3.97	7.991	<0.001
Professional development	40	15.69 ± 3.16	21.11 ± 3.25	7.562	<0.001
Education/consultation	40	19.87 ± 3.33	25.36 ± 3.51	7.176	<0.001

### The implementation effect of “Internet + medical” mode three-level enteral nutrition nursing system

After 1 week of nursing, NRS2002 scores decreased and levels of albumin and hemoglobin increased in both groups of patients (*P* < 0.05). However, the NRS2002 scores in the study group were lower than in the control group, and the levels of albumin and hemoglobin were higher than in the control group (*P* < 0.05; see Table 3).

**Table 3:** Implementation effect of “Internet + medical” enteral nutrition nursing system ($\bar{x}$ ± s).

**Group**	**Cases**	**NRS2002 score (scores)**		**Albumin (g/L)**		**Hemoglobin (g/L)**	
		**Before nursing**	**After 1 week of nursing**	**Before nursing**	**After 1 week of nursing**	**Before nursing**	**After 1 week of nursing**
Study group	40	4.22 ± 1.03	2.89 ± 0.75^a^	32.13 ± 3.78	39.89 ± 3.21^a^	106.35 ± 12.86	119.57 ± 8.78^a^
Control group	40	4.12 ± 1.39	3.25 ± 0.82^a^	33.06 ± 3.92	36.25 ± 3.45^a^	107.01 ± 10.52	113.66 ± 9.55^a^
*t*		0.365	2.049	1.080	4.885	0.251	2.880
*P*		0.716	0.044	0.283	0.001	0.802	0.005

Compared with before nursing,

a P < 0.05.

## Discussion

In clinical practice, after nutritional treatment and nutritional formula are determined, nurses complete practical work like catheter placement and maintenance, nutrient solution configuration and infusion, prevention of complications related to enteral nutrition, publicity and education of patients with relevant knowledge, nutritional monitoring, and establishment and management of information databases (9). Therefore, the nursing activities of nurses are closely related to the implementation results of enteral nutrition. Furthermore, nurses' specialized nursing knowledge and technology largely determine whether enteral nutrition can be carried out smoothly, as well as influencing the therapeutic effect of enteral nutrition.

Primary hospitals are an important hub of China's health service network. However, a previous report highlighted that, due to the limitation of medical resources, the nutritional support work of primary hospitals has not attracted attention below the national level. In addition, due to old nutritional concepts, the delayed updating of knowledge, insufficient nursing talent, and a lack of unified nutritional support nursing quality standards, it is difficult to carry out nutritional support in clinical practice (10, 11). In addition, medical staff lack knowledge of nutritional support, and there are still misunderstandings about enteral nutrition treatment and nursing (12). Therefore, it is vital that a group of professional nutrition support nurses are trained to guide clinical practice, standardize the quality standards of nutrition support nursing, and improve the level of specialist nutrition support nursing.

The establishment of an “Internet + medical” mode three-level enteral nutrition nursing system is the inevitable result of the rapid development of Internet technology. At present, the system is widely employed in clinical practice and has achieved remarkable results (13–15). In the present study, an “Internet + medical” mode three-level enteral nutrition nursing system was developed to guide the implementation of nutrition training and clinical nutrition nursing in county-level hospitals, achieving good results. In terms of the training of nurses, the results of the present study revealed that, after training in the nutrition nursing system, nurses' cognition and behavior scores in enteral nutrition safety nursing were higher than those before training, the scores of various items on the CIRN scale were higher than those before the training, and the differences were statistically significant (*P* < 0.05). This suggests that the “Internet + medical” mode three-level enteral nutrition nursing system could significantly improve nurses' cognition and comprehensive ability in enteral nutrition. The results are consistent with those reported in the study conducted by Xu et al. (16, 17). In terms of the effect of nursing practice on patients, the results of the present study revealed that, after 1 week of nursing, NRS2002 scores decreased and levels of albumin and hemoglobin increased in both groups (*P* < 0.05), with the NRS2002 scores in the study group being significantly lower than those in the control group, and the levels of albumin and hemoglobin in the study group being significantly higher than those in the control group. This suggests that the “Internet + medical” mode three-level enteral nutrition nursing system can reduce the nutritional risk of patients treated with enteral nutrition and improve their nutritional status. There are two aspects of training using the “Internet + medical” mode three-level enteral nutrition nursing system. First, using the Internet, mobile communication equipment, and a cloud platform, online training was delivered to nurses in a planned way, improving the nurses' cognition of enteral nutrition. Second, offline technical training, team building, and practical guidance realized the three-level linkage and technical guidance of specialist enteral nutrition nursing, further improving the specialized enteral nutrition nursing level of nurses in grass-roots hospitals and continuously improving nursing quality and ensuring the safety of nutritional nursing.

## Conclusion

The construction of an “Internet + medical” mode three-level enteral nutrition nursing system and the implementation of systematic clinical training and enteral nutrition management can effectively improve the cognition level and comprehensive nursing ability of medical staff in the field of enteral nutrition nursing. The clinical application effect is satisfactory, and the system is worthy of clinical popularization.

## Data availability statement

The original contributions presented in the study are included in the article/supplementary material, further inquiries can be directed to the corresponding authors.

## Ethics statement

The studies involving human participants were reviewed and approved by Ethics Committee of The Second Affiliated Hospital of Nanchang University. The patients/participants provided their written informed consent to participate in this study.

## Author contributions

Conception and design of the research, obtaining financing, and writing of the manuscript: MH. Acquisition of data and analysis and interpretation of the data: F-TX. Statistical analysis: YL. Critical revision of the manuscript for intellectual content: J-MX. All authors read and approved the final draft.

## Funding

The work was supported by Science and Technology Program of Jiangxi Health Commission (No. 202210515).

## Conflict of interest

The authors declare that the research was conducted in the absence of any commercial or financial relationships that could be construed as a potential conflict of interest.

## Publisher's note

All claims expressed in this article are solely those of the authors and do not necessarily represent those of their affiliated organizations, or those of the publisher, the editors and the reviewers. Any product that may be evaluated in this article, or claim that may be made by its manufacturer, is not guaranteed or endorsed by the publisher.
