# Corrigendum: Construction of a three-level enteral nutrition nursing system under the “Internet + medical” mode and an evaluation of its effect in clinical application

**DOI:** 10.3389/fpubh.2022.1081571

**Published:** 2022-11-30

**Authors:** Min Hu, Yan Ling, Fang-Ting Xiong, Jian-Mei Xu

**Affiliations:** ^1^Department of Neurology, The Second Affiliated Hospital of Nanchang University, Nanchang, China; ^2^Department of Neurosurgery, The Second Affiliated Hospital of Nanchang University, Nanchang, China

**Keywords:** Internet, medical care, enteral nutrition, management platform, nursing

In the published article, there was an error. Due to an error during production, references in the text relating some tables were incorrect.

A correction has been made to **Abstract**, “*Methods*.” This sentence previously stated:

“A total of 40 nurses from four primary and secondary hospitals in Jiangxi Province and 100 patients treated with…”

The corrected sentence appears below:

“A total of 40 nurses from four primary and secondary hospitals in Jiangxi Province and 80 patients treated with…”

A correction has been made to **Subjects and methods**, “*Patients*,” paragraph 1–4. This sentence previously stated:

“A total of 100 patients in need of enteral nutrition treatment admitted from January 2020 to December 2021 were selected as the research objects. These data were obtained from four primary and secondary hospitals.

Inclusion criteria: (1) Patient age >18 years; (2) indication of enteral nutrition; (3) nutritional risk screening 2002 (NRS2002) score >3; (4) normal cognitive ability; (5) hospital stay >1 week.

Exclusion criteria: (1) The presence of intestinal diseases, such as intestinal ischemia, intestinal obstruction, and intestinal perforation; (2) patients with very severe craniocerebral injury or patients at a terminal stage of disease with a short life expectancy.

A total of 50 patients from January to December 2020 were selected as the control group, and 50 patients from January to December 2021 were selected as the study group. There were 25 male patients and 25 female patients in the control group, with an age range of 18–75 years (average age 43.58 ± 10.53 years). The cases in the control group included 15 cases of cerebral infarction, 12 cases of acute pancreatitis, 12 cases of tumor, and seven cases of craniocerebral trauma. There were 27 female patients and 23 male patients in the study group, with an age range of 18–75 years (average age 44.25 ± 9.86 years). The cases in the study group included 13 cases of cerebral infarction, 13 cases of tumor, 12 cases of craniocerebral trauma, and 12 cases of acute pancreatitis. The general data of the two groups were comparable (*P* > 0.05).”

The corrected sentence appears below:

“A total of 80 patients in need of enteral nutrition treatment admitted from January 2020 to December 2021 were selected as the research objects. These data were obtained from four primary and secondary hospitals.

Inclusion criteria: (1) Patient age >18 years; (2) indication of enteral nutrition; (3) nutritional risk screening 2002 (NRS2002) score >3; (4) normal cognitive ability; (5) hospital stay >1 week.

Exclusion criteria: (1) The presence of intestinal diseases, such as intestinal ischemia, intestinal obstruction, and intestinal perforation; (2) patients with very severe craniocerebral injury or patients at a terminal stage of disease with a short life expectancy.

A total of 40 patients from January to December 2020 were selected as the control group, and 40 patients from January to December 2021 were selected as the study group. There were 21 male patients and 19 female patients in the control group, with an age range of 18–75 years (average age 43.58 ± 10.53 years). The cases in the control group included 15 cases of cerebral infarction, 12 cases of acute pancreatitis, 6 cases of tumor, and seven cases of craniocerebral trauma. There were 23 female patients and 17 male patients in the study group, with an age range of 18–75 years (average age 44.25 ± 9.86 years). The cases in the study group included 13 cases of cerebral infarction, 3 cases of tumor, 12 cases of craniocerebral trauma, and 12 cases of acute pancreatitis. The general data of the two groups were comparable (*P* > 0.05).”

The authors apologize for these errors and state that this does not change the scientific conclusions of the article in any way. The original article has been updated.

## Publisher's note

All claims expressed in this article are solely those of the authors and do not necessarily represent those of their affiliated organizations, or those of the publisher, the editors and the reviewers. Any product that may be evaluated in this article, or claim that may be made by its manufacturer, is not guaranteed or endorsed by the publisher.

